# The fight for power: historical women’s movements of Russia and Great Britain in comparison

**DOI:** 10.3389/fsoc.2023.1101380

**Published:** 2023-11-20

**Authors:** Eva Maria Hinterhuber, Jana Günther

**Affiliations:** ^1^Faculty of Society and Economics, Rhine-Waal University of Applied Sciences, Kleve, Germany; ^2^Department of Social Work, Evangelische Hochschule Darmstadt – University of Applied Sciences, Darmstadt, Germany

**Keywords:** women’s movement, protest movements, social movements, women’s history, power relations, gender politics, gender relations in history

## Abstract

In the second half of the 19th century, women began to organize worldwide to achieve the goal of gender equality. National women’s movements emerged and were followed somewhat later by the first transnational political mobilization of women on a larger scale. The range of topics that were on the national and international agenda included, alongside the access to education and the enforcement of equal civil rights, as well as the fight for political participation, with the women’s right to vote taking center stage.^1^ The political, social, and cultural contexts, in which women raised their voices, varied. On the national level, female activists often had conflicting positions and their strategies reflected a wide spectrum; the chosen forms and the course of the protest, on the other hand, showed similarities.

## 1 The fight for power: an introduction

A systematic comparison^2^ of selected historical women’s movements presents an opportunity to map out the differences and parallels regarding framework conditions and starting points, as well as point out the continuities and gaps between historical and current women’s movements. With the early Russian and British women’s movements of the turn of the century, the choice fell to two movements that were subject to unique historical and political circumstances. Therefore, we are using the “most-different-case selection rationale” (Beckwith, 2005, 419), which is relatively rare in comparative studies of women’s movements.^3^

The central issue of our comparative case study is how early women’s movements acted in their specific national contexts, which external and internal power relations existed, how the women’s movements themselves defined such relations, and whether these relations could be utilized for empowerment or were a reason for failure of the movement. For that purpose, we develop firstly a theoretical interpretation framework for the analysis of power and empowerment in (historical) women’s movements. Against this background, the history of the early British and Russian women’s movement will be described. Based on this, there will be a systematic comparison exploring parallels and differences, and drawing conclusions – including, but not limited to, continued challenges of gender based political mobilization.

## 2 Theoretical framework of interpretation: power ‘over’, power ‘with’, and power ‘to’

It is the ability to act and to embark on something new that turns people into political beings (Arendt, 1972, 179). “[T]o get together with [...] peers, to act in concert, and to reach out for goals and enterprises” (Arendt, 1972, translation by the authors) is what refers to the creative forces of individuals in their societal contexts and to the correlation between action and power. Social movements – as an association of “political beings” in the Arendtian sense – undertake the attempt to jointly influence social conditions and to advance social change (Roth and Rucht, 2008, 13).

Therefore, women’s movements as social movements cannot be dealt with without addressing the concept of power. In the context of women’s movement(s) different manifestations of power are important: the spectrum ranges from ‘power over (someone or something)’, through ‘power with’ in cooperation with others to ‘power to (something)’ (Rowlands, 1998, 14) as the “capacity to reach a goal and show resistance” (Göhler, 2012, 255; translation by the authors). Each of these forms also has its drawbacks; additionally, in this understanding, power is not a limited resource, meaning a gain in one form may also lead to a gain in another (comp. Göhler, 2012).

Women’s movements are considered to be agents of a change that was won or, respectively, that is to be won in terms of equality of the genders. Women’s movements result from and act in specifically shaped hierarchical gender relations. Already in the previous centuries, they indicated complex, heterogeneous relations of domination and subordination and used them as a starting point for various kinds of gender political mobilization. In this context of gender relations as power relations, it is the ‘power over something or someone’ that is meant, such form of power that is in the position to exercise control over actions or choices of others by the means of one person or a group, for example according to Weber (1972, 28), or Robert Dahl: “A has power over B to the extent that he [*sic*] can get B to do something that he otherwise would not do” (Dahl, 1957, 209). From a gendered perspective, women’s movements (including those of previous generations) mobilized against the overt practice of ‘power over’ – e.g., as physical coercion – as well as against its subtler form, that is internalization of control, which makes violence obsolete.

Gender political mobilization against existing gendered power relations shows a further expression of power in the context of (not only) historical women’s movements: the ‘power with’ – to act in solidarity with others, the fact that the chances to make impact are generally higher for a group than for separate individuals (Rowlands, 1998, 14). This is where the type of power already mentioned comes into play in Arendt’s sense (interpreted positively in this context), power as “the human ability not just to act but to act in concert” (Arendt, 1972, 143, translated by the authors). In addition to dominant gender regimes, women’s movements anchored in specific historical, political, economic, social and cultural circumstances characterized by other dimensions of social injustice. The specific power constellations that result from the intersection of those factors form the field in which women’s movements operate. To gain power, alliances are also made with hegemonic actors; power relations within women’s movements can be altered and tensions may arise. Co-operation with external actors can lead to successes; however, they also harbor the danger of co-optation and erosion of positions and demands.

Consequently, women’s movements differ according to their national contexts, although an international comparison of their specific goals and ways of mobilizing shows considerable parallels.^4^ Further, the history of women’s movements is not only heterogeneous from a national perspective. There are similar hints that within national movements, the “putty” (Haunss, 2011, 36) of social movements, meaning the specific forms of collective identity (Melucci, 1989; Eder, 2011), are articulated in various ways. Seen from such a perspective, women’s movements are not only producers or carriers of social struggles in gendering and gendered power relations. They themselves become a place where power relations reflect and replicate. The ‘putty’ of identity politics, in particular the appeal to gender identity, consequently provides a trigger for social explosive power already in the early feminist movements. That is, other societally relevant social relations of injustice are targeted and express themselves through demands, forms of mobilization and organization. Where people act in concert (Arendt, 1972, 179), i.e., at the level of resistance protest, it comes even to fighting for power to act, but also for power of interpretation.

Conflicts and factions are, in our conceptualization, an inherent part of historical women’s movements, in which exclusions are established along with inequality and hegemonic gender concepts. These lines of conflict, however, also enable the broadening of the protest spectrum, the establishment of new organizations and solidarization as well as the setting of new goals. This is where ‘power to’ plays a role, the power that is generative and productive (Hartsock, 1983, 223) – “power as potential, as energy […], which aside from the own self can be bestowed upon others, and which is expressed in forms of mutual transfer of power” (Albrecht, 1999, 112; translation by the authors). To utilize these forms of power, gender political mobilization under the circumstances of the above-mentioned conflicts depends on continuous processes of deliberation and (self)communication within women’s movement(s): in the words of Nicholson (1995, 62) it is always also a kind of internal politics of coalition: “The coalition politics of such movement[s] would be formulated in the same way as coalition politics in general are formulated, as either comprised of lists of demands articulated at a certain abstract level to include diversity, or as comprised of specific demands that diverse groups temporarily unite around” (Nicholson, 1995, 62). The necessity of coalitions between vastly different groups in order to achieve (generative) power together also applies to historical women’s movements. The (self-) empowerment to begin ‘something new’ is a complex collective act.

The aim of the following parts is to describe the struggles of historical Russian and British women’s movements against gendered power relations and with that, ‘power over’, to present whether and how they acted together and in solidarity (Göhler, 2012, 255) in the sense of ‘power with’ as well as how they managed to reach new goals (‘power to’; Göhler, 2012).

## 3 Historical women’s movements in Russia and great Britain between power and empowerment

At first, outlooks on the histories of the British and the Russian women’s movements between the fin the siècle and 1917 are presented. In a brief review, the first protests against the hierarchical gender order in the first half of the 19th century will be discussed, and then the mobilization of gender politics in the period between 1905 and 1917 will be examined: This period can be identified as the genesis of the British and Russian women’s movements.

The late 19th and the beginning of the 20th century have been marked by massive social tensions and historical events in both countries, followed by incisive societal, political and economic changes. In connection with the social and political upheavals of the time, the solidification of the two women’s movements, their increasing visibility on a national level as well as in an international context, the establishment of new organizations, their forms of protest and objectives as well as the internal differentiation within the British and Russian women’s movements are traced.

### 3.1 Russia: socialism vs. feminism

Russia looks back on a traditional and comparatively longstanding women’s movement. Its start is usually identified in the year 1863, and linked to the publication of Nikolay G. Chernyshevsky’s utopian novel “What is to be done?’’ (Chernyshevsky, 1900). Widely received within existing revolutionary movements – “from the Narodniki^5^ to Russian social democracy to the Bolsheviki^6^” (Möbius, 2015, 159; translation by the authors) – “What is to be done?” is considered a key text as well for the emerging Russian women’s movement (Möbius, 2015), not least because the author closely tied his vision of a future society to gender equality.

The existent gender regime of czarist Russia in the 18 and 19th century was far from coming close to this, instead, it rather reflected the absolutist character of the political regime (Stites, 1978). The subordination of women was enshrined in law, particularly within the moral code Domostroy and the Swod zakonov collection of laws (for details see Köbberling, 1993, 10). However, this is another case in which the actual consequences could vary: On the one hand, there was the unrestricted power of the husband in the countryside (Köbberling, 1993, 12), on the other, early attempts within the Intelligentsiya^7^ to establish more leeway within lived gender relations. This was accomplished through subversive practices such as fake marriages (for which, after its publication, the novel “What is to be done?” often provided a point of reference and a kind of script).

From the middle of the 19th century, made possible by political reforms^8^ and inspired by emancipatory social utopias such as those of Chernyshevsky, it is possible to speak of the formation of a women’s movement in Russia. This was not a movement of the masses, but primarily supported by the Intelligentsiya within conurbations such as the metropolises of Moscow and St. Petersburg (Köbberling, 1993, 13). From the beginning, the women’s movement consisted of different currents: feminists, nihilists and radicals.

The first primarily directed their efforts toward social services, associations, and conferences; they examined family, education, and economy through a gendered lens and aimed for reforms, not a change of the system (Köbberling, 1993). At no point, the feminists constituted a homogenous movement; instead, they were made up of a variety of sometimes contradictory strands (Köbberling, 1993, 15). Nihilism, on the other hand, strived for total freedom in an anarchist sense (Köbberling, 1993, 14), which was linked to demands for a right to sexual self-determination and equal educational opportunities for women and men. Goals were meant to be achieved mainly through alternative forms of living, rather than political means.^9^

Repressions after the fatal attack on Alexander II in the second half of the 1880s pushed the movement(s) into illegality, which led to a radicalization of the protagonists (Köbberling, 1993). Among the so-called radicals, who aimed to abolish Czarism through political murder – a goal which they achieved after the attack on Alexander II in 1881 – were numerous prominent women. Topics of gender equality took a back seat, leading Köbberling (1993, 15) to the verdict that they ceased to be a “women’s movement in the proper sense” (Köbberling, 1993, translation by the authors). Since the “tyrannicide” of the Czar was not followed by a democratic system, but by the continued existence of an autocratic system, the movement eventually to a large extent ceased to exist (comp. Köbberling, 1993) For the women’s movement at large, it is also possible to speak of a “break” from 1881 to 1905.

From the Russian Revolution on 1905 to 1907,^10^ carried by a “revolutionary movement rising and declining in several great waves” (Stökl, 1990, 596; translation by the authors), the women’s movement emerged visibly (Iukina, 2013, 38ff.; Ruthchild, 2010). A socialist women’s movement and a feminist suffrage movement following the British suffragettes proved the most visible (Köbberling, 1993, 16; Racioppi and See, 1997, 20; Edmondson, 2001, 155–156).

On the feminists’ side, the focus lay on social affairs and therefore, among other things, on education and upbringing and the fight against prostitution. One of the best-known associations is the “Russian Benevolent Society of Women” founded in St. Petersburg as early as 1895 (Köbberling, 1993, 16; Garstenauer, 2010, 79). The socialists, on the other hand, referred to Nadezhda Krupskaya’s newspaper “The Woman Worker” (1900), in which Lenin’s life companion rejected “all ‘feminist’ solutions” to the women’s question (Köbberling, 1993, 19; translation by the authors), as well as August Bebel’s “Women under Socialism” (Bebel, 1879). The central point was the (main) contradiction between capital and labor that could be overcome through socialism, resolving the “side issue” of the inferior social position of women.

When after the “Bloody Sunday” on January, 9th (22nd) 1905^11^ (Stökl, 1990, 596), a brutal suppression of worker protesters of all genders (comp. Also Alpern-Engel, 2003, 254) before the Winter Palace of St. Petersburg leading to many casualties, authorities were pressured into new reforms, including the freedom of association, new women’s associations were formed. Among these were the “Union for Women’s Equality” in February 1905 (Garstenauer, 2010, 79; Köbberling, 1993, 17) that united a broad spectrum to fight for women’s rights: liberal feminists, social democrats, socialists and social revolutionaries. Alpern-Engel (2003, 255) even goes so far to assess that “[t]heir common ground of opposition to autocracy led members of the Union for Women’s Equality to collaborate with liberal and leftist men far more than feminists did elsewhere in Europe or in the United States” and emphasizes, that the Union aimed at speaking “on behalf of all women, and not just the women of the middle class who were their main constituency” (Alpern-Engel, 2003).

In 1907, the Union already counted 12,000 members. It advocated the rights of worker and farmer women, as well as coeducation. Its demands were addressed toward both opposition and government; to the former, it appealed to see the women’s question as an integral part, from the latter, it demanded social and especially political rights for women (Köbberling, 1993, 17). Despite the official interdiction of promoting women’s suffrage, the aforementioned “Russian Benevolent Society of Women” opened a so-called “suffrage department” in 1905, while in the same year a “Progressive Women’s Party” was formed. Indeed, the reforms achieved by the revolutionary upheavals included the convocation of the parliament which had limited rights, land reforms and on December 11/24th, 1905, the enactment of a “general” suffrage.^12^ From the women’s movement’s perspective, this election law proved disappointing, since it granted suffrage to women only in very few cases, bound to specific pecuniary and family circumstances, where the woman acted as a representative.

Three years later, the “First All-Russian Women Congress” was held in St. Petersburg with approx. 1,000 participants (Köbberling, 1993, 18; Godel, 2002, 298; Alpern-Engel, 2003, 256ff.). Though first boycotting the congress, the socialists then decided to send a delegation headed by Alexandra Kollontai.^13^ Her position that the solution of the “woman’s question” was secondary to the overcoming of class antagonism was met with considerable protest. The resolution drafted eventually contained “demands for work safety and maternity protection, right to education and reform of divorce rights” as well as “the universal, equal, free and secret suffrage” (Köbberling, 1993, 18; translation by the authors). The differences surfacing during the congress “within the feminist movement, but even more profound between middle-class feminists and the few working women in attendance” (Alpern-Engel, 2003, 256), however, led to the end of the “Union for Women’s Equality” (comp. e.g. Garstenauer, 2010, 79) which had provided common ground for different currents within the women’s movement (Köbberling, 1993, 19).^14^

On the socialists’ side, certain accomplishments could be noted within their own political camp. Firstly, from 1913 on, the Russian Social Democratic Labor Party (RSDLP) intensified the recruitment of women for their political agenda. On the 8th of March of the same year, socialist women managed to celebrate the International Women’s Day for the first time. Beyond that, secondly, the demand to open women’s departments was granted, even though these had only little influence. Both parts of the RSDLP, split since 1903, published women’s magazines, on the Bolshevik side the “Rabotnitsa” (engl. “The Woman Worker”), on the Menshevik side the “Listok Rabotnitsy” (engl. “The Woman Worker’s Paper”; comp. Alpern-Engel, 2003, 159).

The differences within the Russian women’s movement became even more apparent with the onset of the First World War in 1914. “Between June 1907 and the outbreak of World War I, the women’s movement splintered and lost membership and momentum” (Alpern-Engel, 2003, 256). On the feminist spectrum, patriotic groups openly supported the war, among them the “All-Russian League for Women’s Equal Rights.” The vast majority – even of those feminists, who did not support it – saw it as an opportunity to change the gender relations, not least to introduce the women’s suffrage (Köbberling, 1993, 19; Garstenauer, 2010, 80). The opening of universities to women in 1916 has been attributed to the bourgeois women’s movement, without considering the military-economic causes (Köbberling, 1993, 20f.).

The socialist part of the women’s movement, however, opposed the war by a majority and showed pacifist and anti-militarist involvement, even on an international level. Together with other socialist women’s movements, it assembled at the International Socialist Women’s Conference in Bern in 1915 and the 1915 International Congress of Women of the International Committee of Women for Permanent Peace in The Hague. Alpern-Engel, thus, relates the divergent attitudes toward the war mainly to class issues, according to which “[w]omen of the upper class had enthusiastically supported the war effort” (2003, 261) in the hope of an increase in equal participation, whereas working-class women could not expect neither an improvement of their material well-being, nor more peaceful times. Military defeats as well as catastrophic supply conditions led to insurrections against the ruling Czar Nicholas II for the population, starting from 1916, which led to his abdication and assassination in 1917 (comp. Schröder, 2010). And it was women’s demonstrations that “took place on February, 23rd (March, 8th) [1917] on the occasion of the Socialist Women’s Day,” that “were going to be the beginning of the sudden end of the Russian czarism” (Stökl, 1990, 635; translation by the authors). Strikes and demonstrations of politically unorganized worker women eventually catalyzed the February Revolution (Köbberling, 1993, 23; Godel, 2002: 299). After the czar’s resignation on March, 3^rd^ (16^th^), a provisional government took charge of the government affairs (Stökl, 1990, 639 and 640ff.); “Russia had become a republic overnight” (Stökl, 1990, 639; translation by the authors).

In hindsight, it appears that feminist organizations seized the opportunities arising at this point. Still in March 1917, a major demonstration for the introduction of women’s suffrage was held by 40,000 participants of all genders (Köbberling, 1993, 24). A merger of several feminist organizations into a “National Women’s Council” got recognized by the government in May of the same year. During the 8 months of its reign, the provisional government introduced the women’s suffrage, giving Russia a historically pioneering role by international comparison, and established the principle of equal pay; even the opening of women’s universities was planned.

Assessing these accomplishments in women’s politics, further differences between feminists and socialists manifested: the latter “considered a true liberation of women only possible through the abolition of private ownership of means of production” (Köbberling, 1993, 25; translation by the authors) and rejected mere reforms, even within a new political system. The conflict escalated further when a delegation of Bolshevik women ostentatiously left the All-Russian Women’s Congress in April 1917, a further cooperation seemed impossible (Fieseler, 1993, 169).

After the October Revolution (Stökl, 1990, 646) and coming into power by the Bolsheviks, ushering at the beginning of the Soviet era, the feminists definitely got on the defensive. Feminism, a former political fighting slogan, became stigmatized.^15^ The growing political pressure on feminist figures led to an increase in emigration to Europe (Köbberling, 1993, 26). “Starting from late 1917, the political activity of women was limited to the Communist Party – any other approaches had been eliminated very soon” (Köbberling, 1993, 27; translation by the authors). During the years following the Bolshevik seize of power, a significant “equal treatment from above” has been imposed: among other things, liberalization of marriage and divorce laws, introduction of coeducation, legalization of abortions within certain limits, but most importantly, the accessibility of work spaces to women and their position within these has been profoundly reformed, aiming at the socialization of domestic work (Köbberling, 1993, 28ff.; regarding the Soviet gender politics: Attwood, 1990; Evans Clements, 1991; Rosenbaum, 1991; Schmitt, 1997).

Köbberling (1993) summarizes the differences between socialists and feminists in the women’s movement along theoretical, organizational and practically-political questions: on a theoretical level, feminists strived for reforms, without questioning the political system *per se*, while socialists aimed for a system change; feminists pursued the objectives of women’s politics in different organizations, whereas socialists organized themselves under the umbrella of the RSDLP; and finally, feminists hoped for an instrumentalization of the war in favor of women’s political aims, thus supporting the war, in contrary to the socialists, who opposed it. These differences had different significance at different times: “When, around the turn of the century, a socialist women’s movement started to emerge for the first time, a limited cooperation of feminists in the interest of achieving certain goals (such as women’s suffrage) would absolutely have been possible” (Köbberling, 1993, 22; translation by the authors). And indeed, “meaningful collaboration between socialist organizers and feminists did occur during these years, revealing a greater permeability in the boundaries between Russian socialism and feminism than is generally recognized” (Norton, 2011, 237, referring to Ruthchild, 2010).

To strive for goals of women’s politics from different – feminist and social revolutionary – standpoints, however, was no longer possible after the Bolshevik takeover. And, putting aside the achievements, it has to be remarked that the strategy of “equality from above” since 1917 has also led to the co-optation and destruction of the proletarian women’s movement, which had existed since 1905 (Ruthchild, 2010, 43ff.).

How, in comparison, did the British women’s movement act within its specific historical and political context? Which external and internal distributions of power influenced it, and how could the movement influence them, in turn? Which potentials for emancipation did it have, and where did it meet a greater risk of failure?

### 3.2 Great Britain: constitutionalism vs. militancy

In Great Britain – in contrast to its Russian counterpart – the women’s movement succumbed to the changing political and economic conditions that resulted from the Glorious Revolution of 1,688 and the emerging industrialization. Even though increasing democratization and disempowering of the absolute monarchy were interrupted by restorative phases, the British had a long tradition of parliamentarism. This was characterized, among other things by the political emancipation of the bourgeoisie successive to the reformist politics (Karl, 2011, 62). Women explicitly joined in with the demands for political participation. In “Vindication of the Rights of Women” (Wollstonecraft, 1792), Mary Wollstonecraft highlighted the systemic exclusion of women and rose to become one of the founders of modern feminism (Caine, 1997, 24). Nevertheless, Great Britain in the Victorian era continued to keep women confined to “the domestic hearth” (de Beauvoir, 2000, 172; translated by the authors). Consequently, Jane Austen had to hide “in order to be able to write” and “much courage and an extraordinary fate” was required in order to become “a George Elliot or Emily Brontë” (de Beauvoir, 2000). In addition to this, Queen Victoria herself believed that women who asked for suffrage should simply be subject to the lash (Lloyd, 1970, 5).

The earliest feminist activists found a political occupation in the Owenist^16^ union’s movement (Hannam, 1995, 219). Thus, for instance, the Owenists/early Socialists William Thompson and Anna Doyle Wheeler insisted in their “Appeal of One-half of the Human Race, Women, Against the Pretensions of the Other Half, Men, to Retain Them in Political, and Thence in Civil and Domestic Slavery” (Thompson and Wheeler, 1825), that women were “more in need of political rights than any other portion of human beings” (Caine, 1997, 59). The emerging worker’s movement itself, along with its counterparts in other European countries, had an ambivalent stance toward the women’s question. As in the Russian example, this mirrored the idea that with the resolution of class struggle, gender inequality would also be resolved.

In the United Kingdom, women of the working class became members of the Trade Unions (Thompson, 1981, 175). They also organized against the restrictive New Poor Laws, which promoted social division. This meant that public resistance, such as the bread riots and the worker’s struggles, were carried by female workers and wives of workers. Consequently, they were more visible to the public eye than the women of the middle and upper classes (Hannam, 1995, 219; Rowbotham, 1980, 86). The increasing, at times vehemently and violently fought struggles led to significant reforms. However, these reforms benefited chiefly middle-class men.

The independent “Chartist party” was formed following the Great Reform Acts of 1832, which instituted voting rights based on property and thereby excluded a large portion of the working class, as well as women as a whole (Engels, 1891, 100). “Women played their role in the general upheaval of Chartist politics. They participated in protests and actions against the police, the established church, against corporate exploitation and the encroachments of the state” (Thompson, 1981, 177). Although in its beginnings, the Chartist movement had demanded suffrage for women (West, 1920, 11), it eventually abandoned this demand in favor of general voting rights for men (West, 1920, 79). The strategic assumption was, that after full voting rights for men had been secured, “the expansion of political rights to women would follow on the basis of natural justice” (Thompson, 1981, 179). This logic was later echoed by the women’s movement in its demands for suffrage, in particular to justify the exclusion of certain classes (and thereby poorer, mostly working-class women).

The Charter Movement is particularly relevant to the construction and specifics of the history of women’s movements in Britain because it represents the earliest documentable organizations and strategic mobilizations of women. Not least among these was the foundation of the first organization for women’s suffrage – the Sheffield Female Political Association – in 1851, by the Chartist Anne Knight. She justified her activism as follows: “NEVER [!] will the nations of the earth be well governed, until both sexes, and all parties, are fairly represented, and have an influence, a voice, and a hand in the enactment and administration of the laws. […]” (Knight, 1847; as cited in Blackburn, 1902, 19).

Apart from fighting for political power, women in the middle of the 19th century also protested the double-standards of Victorian society regarding moral questions. Women of the upper classes, who were active in social reforms and philanthropic causes, had to face the problem that “men of their own class” were the beneficiaries of poverty in women of the working class, while they themselves were trying to “cure the social consequences of prostitution” (Rowbotham, 1980, 71). The “ease with which men of the middle class regarded the prostitution of working women” ironically contrasted with their “concern for the virginity of their own daughters” (Rowbotham, 1980, 72). With the spread of sexually transmitted diseases and the institution of the Contagious Disease Acts, this gender^17^- and class^18^-based exploitation was enshrined in law. The protests against these laws are commonly associated with activist Josephine Butler, who organized a broad protest of her Ladies National Association (LNA; Caine, 1997, 122). The demands for abolition of the laws were supported by the working class (Walkowitz, 1980, 146). This enabled strategic coalitions across class boundaries. The broadly based movement could therefore realize a repeal of the law in 1886 (Lloyd, 1970, 27). This “cross-class solidarity” (Holton, 1996, 36) was then translated – although not without conflicts – into the context of voting rights. As this shows, the thematic spectrum and goals of women’s movements in Russia and the UK show considerable overlap.

Even though since the 1850s campaigns for women’s right to vote had a certain public response and such political figures as John Stuart Mill, Henry Fawcett, and Richard Pankhurst, to name just a few parliamentarians, proposed and supported requests for women’s voting rights in the House of Commons, their advances did not succeed (Blackburn, 1902, 55).

With the foundation of the National Union of Suffrage Societies (NUWSS) in 1897, the suffragette organizations of the British women’s movement managed to create an organizational roof, under which focused organizing for the issue of voting was to take place. The NUWSS – under the leadership of a liberally minded economist Millicent Garrett Fawcett – understood itself as a constitutional organization. The political system was supposed to change on the basis of existing democratic principles. The tactics were limited to the parliamentarian logic of the corresponding lobbying policy that was expected to convince the parties (Holton, 2008, 289). Individual Members of Parliament (MP), who were sympathetic to the cause of the women’s vote, brought in the corresponding Private Member Bills.^19^ The female activists with their petitions were accepted and treated politely (Lloyd, 1970, 46). However, for the most part, the requests were not brought to a vote because of ‘talk out’-practices of the opponents: “In 1890, this happened in favor of a bill about raisins and currants; in 1893 in favor of a bill on the taxation of machines; in 1897 in favor of a bill on vermin and in 1905 in favor of a bill on street lighting” (Schirmacher, 1976, 24, translated by the authors).

The tactic of NUWSS, therefore, did not work out on the level of the Parliament, while at the same time it raised awareness of women’s political disadvantage countrywide through special campaigns. Particularly within the industrial districts in North England, the member organizations managed to mobilize a broad faction of working women for the fight for suffrage. Here, the bourgeois activists met politically active working women and female union members, who enriched the campaigns of the previously decidedly bourgeois suffrage movement with their own tactics.

In the socialist and social democratically shaped industrial centers, a new generation of the women’s movement established itself. After the disappointing experiences of recent years, they used more radical tactics to make themselves heard. With the founding of the Women’s Social and Political Union (WSPU) in 1903 by members of the Independent Labor Party (ILP), the struggle for political determination took a new, militant pace. Contrary to the NUWSS, the WSPU did not accept any male members and wanted to be independent of the party despite its roots (Pankhurst, 1913, 38). Like the NUWSS, however, WSPU demanded only a limited right to vote on the same conditions as men had until that time. Although this form of voting excluded in particular “lower” classes, initially it was supported by the organized workers (Neumann, 1921, 9). Here the British movement succeeded, in the sense of the ‘power with’, in creating a basis for common action, which was not yet historically possible in czarist Russia, which was at the time parliamentary completely uncoined.

However, a goal of achieving restricted women’s voting rights had led to conflicts at international conferences on the issue. So, Fawcett’s request in 1909 at the International Congress of the World Federation for Women’s Voting Rights, London, caused, among other things, irritation among the socialists Alexandra Kollontai and Clara Zetkin: it seemed unacceptable to them that only members who demanded the right to vote on the terms as they were set for the men in the respective countries could be elected to the Union (n. a, 1909, Gleichheit, 270). Consequently, Socialist women were faced with the question whether there would be “voting rights for the female pocketbook of political power for the owning class and the reactionaries, or civil rights for all those of age irrespective of gender and, therefore, the political liberation of women of all classes and a strengthening of democracy” (n. a, 1909, Gleichheit, 276).

In contrast to czarist Russia (and, by the way, also the German Empire), however, the British working class did not consolidate with the same force, since Socialist and Social Democratic movements were not prosecuted and oppressed by those in power, but (at least through their male representatives) incorporated into the parliamentary process. Consequently, the women’s movement managed to draw on the tradition of “cross-class solidarity,” which drew the attention of international delegates at the mass demonstrations (Pappritz, 1909, Centralblatt, 25ff).

However unspectacular the demand for restricted voting rights may seem to be from today’s perspective, the suffragette movement pursued its goal, gaining public attention and with increasing radicalism, as did the British constitutional and voting rights organizations in other countries. The tremendous popularity and success of the marches and demonstrations also illustrates the fact that cross-class alliances for the right to vote have managed – despite the modest goal – to convey strategic demands. The commotion and following arrest of Annie Kenney and Christabel Pankhurst in Manchester, 1905, is considered the first militant act, in which they disrupted an event by Liberals, incited a street protest together with other women, vehemently resisted their arrest and spat at several Bobbies in the process (Pankhurst, 1931, 190).

The narrative of a fighting suffragette, which exists to the present day, was established with– not always beneficial – media attention which was also heated by the militant tactics. The emerging militant movement subsequently succeeded in gradually presenting itself as a modern and radical one through effective public actions, in attracting numerous fellow fighters to its side and playing on the attention of the press and media economy by the means of constant scandal (Günther, 2017). This strongly suggests that the women’s movements successfully shaped public discourse the way they meant to. The topic of the democratic participation of women was discussed in media and had to be negotiated parliamentary on the basis of petitions, deputations and submitted requests. Parades, assemblies, and arrests near the Parliament or houses of well-known politicians were not only critically covered by the press, but also provided the militant activists with many new supporters (Wingerden, 1999, 76). From 1907, the constitutional NUWSS also opened itself up to forms of street protests and shifted its campaigns more intentionally into the public sphere (Rosen, 1974, 79). Fawcett condemned the militancy of the Suffragettes, but admitted in a solidary letter to the Times that they had achieved more for voting rights for women in the past 12 months than the entire movement had within the past 12 years (Fawcett, 1906, as cited in Marlow, 2001, 46).

Not only the Russian women, but also other European women’s movements, such as the German middle-class women’s movement, looked to the British campaign as a model and gatekeeper. The “victory of women’s right to vote” was first expected here, after which the “beginning of triumph would be in the world” (Schleker Marlow, 1909, 4ff.; translation by the authors). Between 1909 and 1911 due to increasing clashes with the police, further arrests and the first case of destruction of public property, the law enforcement reacted with more restrictive measures and tougher prison sentences. Detained suffragettes called out the poor conditions of their imprisonment, went on hunger strikes and fought for their recognition as political prisoners (Wingerden, 1999, 85). The devastating riots in Birmingham that happened during a visit by the Prime Minister and opponent of women’s right to vote, Herbert Asquith (Pugh, 2002, 192) led to the introduction of forced feeding, for the most part in order not to have to release every protester weakened by hunger. The public outrage over that triggered a reaction of solidarity and led to a multi-party alliance of MPs, who worked together on a Conciliation Bill to introduce women’s voting rights. The vote on the bill in 1910 was, however, delayed, which led to further riots, e.g., on the Black Friday (Pankhurst, 1970, 492ff.). As a result, the militant activists relied on violent and destructive acts. Intentional window smashing and arson of mail boxes became regular forms of resistance. Golf courses, museums, churches as well as houses of prominent politicians fell victim to the struggle of the suffragettes. Or in the words of a prominent author and contemporary witness: “And so acids were poured into letter-boxes or upon golf greens, telegraph lines were cut, fire engines were called out on false alarms” (Zangwill, 1916, 309). Parliamentarians, former long-term allies and constitutional women’s vote activists also became the targets of the militants.

This “propaganda of action” ultimately brought the men of the “ruling class” (Rowbotham, 1980, 117; translation by the authors) to their knees. After the bomb attack on the weekend house of the deputy David Lloyd George in 1913 and resulting increasing police repressions against militant women’s movement organizations, the constitutionalists decisively distanced themselves from them, even though they condemned the conditions of imprisonment and force-feeding. The autocratic style of leadership (Thébaud, 2002, 89) along with the stubbornness with which the WSPU’s leadership pushed to bring the Conciliation Bill to the vote again through its militant campaigns, were considered counterproductive (n. a, 1912b, Common Cause, 831). A dispute about the use of violence developed in the WSPU itself, too. Former allies from the working-class movement together with those from the bourgeois and aristocratic spectrum, who had been generously supporting suffragettes organizationally or financially, left the circle of activists after arrests and house searches, although they still publicly supported the cause of women’s right to vote: “Née en 1903 dans le Lancashire, la Women’s Social and Political Union (WSPU) qui, adoptant la stratégie et le type de propagande des socialistes, a réussi à faire du vote une question majeure en Angleterre et ailleurs, s’est. effritée sous l’effet conjugué du cycle violence-répression et de l’autoritarisme des Pankhursts.” (Thébaud, 2002, 89). Finally, the member organizations of the NUWSS were also facing hostility of the general public, the press, and the police. The final failure of the Conciliation Bill also recorded the constitutional flow on the account of the militants (n. a, 1912a, Common Cause, 877).

The “Guerilla Warfare” of the WSPU (Atkinson, 2002, 33) resulted in a “cat-and-mouse game” between the small militant groups of the WSPU and the police. Suffragettes who were on hunger strikes were released, their prison sentences withdrawn. From the parliamentary side, there was an attempt to break this vicious circle in 1913, when the Prisoner’s Temporary Discharge for Ill Health Act, better known as the “Cat and Mouse Act,” was introduced. The law allowed the release of those suffragettes weakened by hunger strikes, to place them under police surveillance, and to arrest them later (Günther, 2009, 112; Rosen, 1974, 193). Although the law was severely criticized and was seen by the public as a way for suffragettes to attack the liberal government, it opened the possibility for the detainees to gradually pull back from the militant struggle (Pugh, 2002, 210).

With the outbreak of the First World War in 1914, the government issued an amnesty for all imprisoned suffragettes (Strachey, 1928, 337). The war, just like in the Russian movement, caused a break in the British women’s movement. A part of the militant wing immediately declared itself patriotic. Christabel and Emmeline Pankhurst, for example, vehemently supported the government’s military policies and renamed the organization magazine “Suffragette” into “Britannia.” In 1917, still in wartime, Emmeline Pankhurst became friends with the Russian general Maria Bočkareva in Petrograd. She was traveling through Russia in order to convince the Soviet government not to withdraw from the war. Bočkareva herself had entered the military in 1914 under the Name Jaschka and was appalled by the diminishing discipline on the frontline following the February Revolution. Under the patronage of Alexei Brussilow, she established the Women’s Battalion of Death in Petersburg (Hacker, 1998, 215ff). Bočkareva and her battalion embodied the military discipline (Hacker, 1998, 215ff) which the WSPU-leadership aimed to also instill in the Suffragettes, following the motto “One Policy, One Programme, One Command “(n. a, 1914, Suffragette, 387).

On the domestic front, the WSPU saw its task now in getting men for the army and women for the home front, the militancy for the right to vote was channeled into a form of national militancy (Wingerden, 1999, 161): “Just as the suffragettes had used military parallels to characterize the suffrage campaign as a war, so they described their own approach to the world war (Wingerden, 1999, 162).

This strategy, however, encountered resistance, and led to splitting of some organizations and formation of others (Rowbotham, 1999, 67f) such as the Independent Women’s Social and Political Union and the Suffragettes of the WSPU (Hanschke, 1990, 34). The NUWSS experienced a similar division: the umbrella organization called on its member organizations to prove themselves ready for showing their capability for citizenship by joining the national military service (Strachey, 1928, 338). Thus, activists of the militant camp such as Sylvia Pankhurst saw themselves united with prominent constitutionalists and pacifists (Rowbotham, 1999, 68).

Women’s rights organizations and women’s trade unions organized the work of women in the factories, not without difficulties but successfully in the long run (Strachey, 1928, 337). Approximately 23,000 health care workers joined the Voluntary Aid Detachments (VADs) on the western front of the British army, and they were recruited from bourgeois and aristocratic circles (Hacker, 1998, 189). Already during the war, the Conference of Electoral Reform worked out a new amendment on the right to electoral law, which allowed for voting rights of women in a restricted form. The law finally came into force in 1918.

## 4 Conclusion: building coalitions, exercising different manifestations of power, and beginning something new

As outlined in the introduction, women’s movements can never be considered homogenous groups, although they tend to be viewed – especially by analyses within political science – as political actors. Women’s movements consist of many currents, sometimes conflicting ones, that at a certain point or over a limited time span pursue similar goals, together or in parallel to one another. They address different expressions of power and depending on their own circumstances reproduce social power relations, for example class relations, within their own ranks. And last but not least, they move in specific historical, political, and other contexts.

This also applies to early women’s movements in Russia and Great Britain. While the British political system was strong and stable and had a long-standing parliamentary tradition, Russia experienced three revolutions and various forms of government, from czarist absolutism through a parliamentary monarchy and transitional governments to a revolutionary dictatorship (Ruthchild, 2010, 5; comp. For consequential effects also Edmondson, 2001, 154, 162–163).^20^

This also has effects on the application of ‘power over’ as the ability to literally limit the choices of women and, as a consequence, on the struggle of historical Russian and British women’s movements against gendered power relations. The Russian women’s movement was directed against the exclusion of women from social, economic, and political participation in absolutist czarism (whether by its reform or overthrow), it stood for civilian (e.g., regarding matrimonial and divorce legislation), social (e.g., the rights of female workers and female farmers) and, last but not least, political rights (first and foremost, the right to vote).

In the historical British women’s movement, the voting right of women stood in the center of the struggle and was supposed to improve the social position of women in all areas. On the one hand, its approach was built on the tradition of choosing the parliamentary path in order to attain political rights. On the other hand, militant groups did not avoid even violent confrontation with the authorities, while being aware of considerable individual risks. Even if forms and the scale of the resistance were not the same throughout the movement, the joint struggle against the exclusion of women from political participation remained intact.

Exercising ‘power with’, in association with others, was a continuous challenge for both the Russian and the British women’s movement. In the Russian women’s movement, the most evident break was between socialists and feminists, while in Great Britain the sharpest conflict line lied between constitutionalists and militant activists. In Russia, the conflict was happening along the intersections of different axes of inequality: feminists saw the starting point for their cause the oppression through gender, while among the socialists the class struggle was perceived as the main motivation (Similar – though not the same – processes can be found in the history of the women’s movement within the German Empire. Here, too, actors of the proletarian and the bourgeois women’s movement were involved in an “obstinate struggle”; Gerhard, 2009, 67).

These class divisions are not found in the British women’s movement on such a scale. The disagreements that arose after the turn of the century were, above all, on the tactics that the movements were supposed to use. Female workers and their organizations demonstrably cooperated for the sake of voting rights for women, although the history of the British women’s movement is as much marked by rifts as by new alliances: “It was not a question of struggle between reactionary middle-class feminists on one side and enlightened Socialists from the working class on the other. The political reality of the Suffragettes’ movement was much more complicated than conventional stereotypes betray” (Rowbotham, 1980, 107, translated by the authors).

Feminist politics, as the history of historical women’s movements points out, is in this sense always a kind of internal coalition policy (Nicholson, 1995, 62), that is, a conscious joint effort undertaken despite existing differences in a certain constellation and often only for a certain period of time in order to pursue a common gender policy goal. In Russia, the movement has been able to unite under the banner of women’s rights for a certain period of time: Under the umbrella of the “Union for Women’s Equality,” representatives of different directions joined forces for a limited period to work together for the sake of shared goals, which was always possible even during marches/demonstrations. In Great Britain as well the right to vote was at the center of the campaign, but even though the main organizations demanded at least restricted women’s voting rights, the unwillingness of the suffragettes to compromise separated different groups from one another. Nevertheless, constitutional suffragettes came to agreement with the organized female workers under the motto “We stand for justice for the workers and women” (n. a, 1913, Common Cause, 360).

A look at the history of different national women’s movements shows that individual actors could often draw on a varying reservoir of (generative) power, based on resources like money, education, time, influence, and networks. This also influenced the internal power plane of the movements, and significantly shaped the relationship between different strands and groups. Especially between Russian working women and the Intelligentsiya, there are demonstrable conflicts around this (comp. Köbberling, 1993, 19). In contrast to this, the WSPU grew out of the movement of working women and the Socialist/Social Democrat milieus in Northern England. Especially in its early years, it recruited its members from this spectrum, however, without the broad and generous support of women from the upper bourgeoisie and the aristocracy, its enormous success in terms of mobilization would not have been possible. The WSPU proved unable to uphold this coalition in the long term and lost a share of its members to constitutional organizations, which only started to systematically address workers’ organizations on the eve of the World War I. The formation of “external” coalitions with other social actors is also relevant for the strengthening of the ‘power with’: here is the social support that the movements experience in their respective contexts plays a role. In this regard, women’s movements happen to also be dependent on the respective prevailing discourse, which strengthens or weakens power positions – even within the movement. In Russia, the divided rejection of the absolutist czarism enabled, despite the already existing differences, a limited amount of joint action. The conflict between feminists and socialists escalated during historical events; against the different political backgrounds in times of massive change, the individual groups were each able to derive some benefit from their proximity to existing (external) power relations. This is how the feminist women’s movement in Russia was able to realize demands central to their agenda in the bourgeois-dominated Provisional Government between the February and October revolutions. With the seizure of power by the Bolsheviks, the socialists gained momentum; the achievements regarding the legal equality of women in the beginning Soviet era can be considered their contribution.

British self-understanding was based on the idea of parliamentarianism and the fight of the population for its political rights. The movement skillfully took advantage of this rhetoric and put its demand into the context of the common history that highlighted Great Britain as the birthplace of parliamentary democracy and the representative institutions (Eustance et al., 2000, 6). That is why other national women’s movement organizations also saw Great Britain as the country which, through its “century-long parliamentary schooling had taken such an uninterrupted rise to ever greater freedom” and therefore would rapidly introduce women’s voting rights (Schleker Marlow, 1909, 4ff.; translation by the authors). Support for demands of gender politics by hegemonic, and therefore powerful, actors can lead to their realization, but may also result in their co-optation, as the example of Russia demonstrates. Successes notwithstanding, Soviet gender equality policy is an example of the aforementioned danger of co-optation and the accompanying erosion of positions and demands: The women’s question had been considered “solved” (see Köbberling, 1993, 57), and gender policies of the following decades were modeled on various topical considerations of demographic and economic nature. Consequently, they often aimed for goals apart from gender equality (see Hinterhuber, 1999, 112, 2012).

The power of the historical movements of women to act (‘power to’), their ability to achieve goals, can be made particularly clear in the struggle against the respective political regime in the certain historical times. In the course of the Russian Revolution (1905–1907), this is shown first by the (newly) founded of women’s organizations (facilitated by the revolution movements that in the czarist regime fought for the right of assembly), and especially in 1917 with the emergence of the Provisional Government of the suffrage was introduced as well as the right to equal wages and access of universities to women. During the Bolsheviks’ seizure of power, profound changes were enabled by “equality from above.”

In the United Kingdom, the generative, productive ‘power to’ manifested itself not least in the ability of the women’s movement to engage in a long-term resistance, to sensitize society and politics using various, partially controversial strategies, and finally gain women’s right to vote by parliamentary means.

After the introduction of political rights, a long-lasting effect is not guaranteed, as the struggles of women’s movements worldwide after the first and second world wars prove. The question about the reproduction of power relations within the women’s movement also came up over and over again, as the critical objections and debates of the early 1970s show: not only issues of class, but also “race” had to be negotiated (Hooks, 1981; Davis, 1986). Even in the present, there are conflict lines along different dimensions of social inequality within the women’s movement that are of great controversy. The overview of historical women’s movements can be used to return to longstanding discussions, to take up old threads, to point out and acknowledge gaps as well as continuities, to establish external and internal power relations, to renew alliances or to enter new coalitions in order to initiate something new in the process of collective empowerment.

## Author contributions

The article is based on a joint research project of JG and EH, which has taken its starting point in an article published in 2017 under the title “Der Kampf um Macht: Historische Frauenbewegungen in Russland und Großbritannien im Vergleich,” in Femina Politica 2017(1), pp. 24–39. In several papers, the authors discussed further questions related to the topic. The article reflects these questions and discussions.

## Footnotes

^2^This article is based on a joint research project of Jana Günther and Eva Maria Hinterhuber, which has taken its starting point in an article published in 2017 under the title “Der Kampf um Macht: Historische Frauenbewegungen in Russland und Großbritannien im Vergleich,” in Femina Politica 2017(1), 24–39. In several papers, the authors discussed further questions related to the topic. The article reflects these questions and discussions. The authors would like to thank Anastasia Kappo-Klevska and Rebecca Knecht for its translation into the English version at hand and Lotta T. Barabasch for the editing. Nonetheless, any mistakes are the responsibility of the authors.

^3^In response to a reluctance toward comparative research concerning historical women’s movements across national borders particularly among historians, especially related to an emphasis on a ‘cultural distinctiveness’ ascribed to Russia, we share Edmondson (2001, 165) assessment: She stresses that “[t]he issue of citizenship and gender, however, needs to be studied across national boundaries, and across differing political systems. It is only then that we may be able to comprehend the full complexity of the relationship between gender and power in human society, as well as arriving at a clearer understanding of the true distinctiveness of any particular culture.”

^4^“Looking at the nineteenth- and twentieth-century movements for gender equality overall, what is as remarkable as the differences is the extent of the similarities and parallel developments, as well as the mutual influences and interactions” (Edmondson, 2001, p.164).

^5^For a detailed description of the Narodniki (“folklorists”; Schröder, 2010; “Friends of the People”; Stökl, 1990, 574) movement, see for example Stökl (574–582).

^6^Schröder calls the Bolshewiki the “radical wing of Russian social democracy” (2010).

^7^Schröder (2010) characterizes the Intelligentsiya as a community of ideas, which aimed for the end of the czarist autocratic political system, criticized social inequality and based its worldview on science.

^8^The downfall of Czarist Russia in the Crimean War (1853–1856) led to political, economic and social reforms under Czar Alexander II (1853–1881), with a particular focus on the liberation of peasants (Stökl, 1990, 536) and the abolition of serfdom. However, the reforms did not prevent the formation of revolutionary movements within the ranks of the Intelligentsiya, such as the Narodniki, who “aimed for the overthrow of Czarism through the enlightenment of the peasant masses” (Schröder, 2010, translation by the authors), partially through violent means: Czar Alexander II died 1881 in a terrorist attack.

^9^Here, too, a novel played a central role: Ivan Turgenev’s “Fathers and Sons” (1862) (Turgenev, 2009).

^10^During the Russian Revolution the intelligentsiya, industrial workers and farmers allied against the czarist regime. The aspired overthrow of the czar could not be achieved, however, a parliament with limited rights (duma) was established (Schröder, 2010).

^11^The difference in the given dates stems from the Julian vs. the Gregorian calendar; the latter was introduced in the Soviet Union and therefore in Russia in 1918.

^12^Compared internationally, the introduction of the suffrage and its extension to women is often divided by decades; taking that into consideration systematically would have large-scale implications for theoretical reflections and empirical studies (Paxton, 2010).

^13^“The Bolsheviks insisted that the woman question must be solved within the framework of socialist revolution, and their (male) leadership accepted the idea of participation in the women’s conference only very reluctantly” (Alpern-Engel, 2003, 257).

^14^Shortly after, in March 1907, it merged into the newly founded “All-Russian League for Women’s Equal Rights” (Garstenauer, 2010, 79), which remained the most influential women’s organization until the October Revolution (Köbberling, 1993, 19).

^15^For more detailed terminology, see Möller (1999, 123, 127).

^16^The Owenist-Socialist union’s movement campaigned, among other things, for a fundamental reorganization of society according to the principles of the Co-Operative movement (Schäffner, 1997, 22f).

^17^Women who were ‘conspicuous’, could, under these laws, be arbitrarily detained and subjected to forced diagnosis and internment (Rowbotham, 1980, 72).

^18^According to Judith R. Walkowitz, the law also showed significant state intervention into the lives of poor people (Walkowitz, 1980, 3).

^19^These bills submitted by individuals could, given the support of the cabinet, become actual laws.

^20^“[W]hile women in countries with representative government fought for decades to win the right to vote, the same struggle took a mere 12 years in autocratic Russia” (Lindenmeyr, 2011, 211 with reference to Ruthchild, 2010).
